# High Satisfaction with Patient-Centered Telemedicine for Hepatitis C Virus Delivered to Substance Users: A Mixed-Methods Study

**DOI:** 10.1089/tmj.2022.0189

**Published:** 2023-03-10

**Authors:** Andrew H. Talal, Elisavet M. Sofikitou, Kejia Wang, Suzanne Dickerson, Urmo Jaanimägi, Marianthi Markatou

**Affiliations:** ^1^ Division of Gastroenterology, Hepatology, and Nutrition, Department of Medicine, University at Buffalo, Buffalo, New York, USA.; ^2^ Department of Biostatistics, School of Public Health and Health Professions, University at Buffalo, Buffalo, New York, USA.; ^3^ Division of Biobehavioral Health and Clinical Sciences, School of Nursing, University at Buffalo, Buffalo, New York, USA.

**Keywords:** telemedicine, hepatitis C virus (HCV), substance users, people with opioid use disorders, mixed methods

## Abstract

**Background:**

While telemedicine may increase health care access for vulnerable populations, data are limited on whether people with opioid use disorder (PWOUD) are satisfied with telemedicine. We assessed PWOUD satisfaction with telemedicine and identified factors that increase telemedicine satisfaction.

**Methods:**

We conducted a mixed-methods study among hepatitis C virus (HCV)-infected persons enrolled at 12 opioid treatment programs (OTPs) throughout New York State. Participants successfully completed HCV treatment either through telemedicine integrated into OTPs (*N* = 238) or through offsite referral (*N* = 106). We evaluated Patient Satisfaction Questionnaire (PSQ) response scores at the initial and final health care encounters and subsequently interviewed telemedicine study participants (*N* = 25) to assess their experiences with telemedicine.

**Results:**

All participants (*N* = 344) successfully completed HCV treatment. We observed no differences in PSQ scores between telemedicine and in-person encounters (98.3% and 98.7% of telemedicine participants provided PSQ scores of satisfied or highly satisfied at each timepoint, respectively). Study participants indicated that attributes associated with high telemedicine encounter satisfaction included: (1) communicating study information, (2) gaining trust, and (3) delivering patient-centered care. Participants weighted “General Satisfaction” and “Time Spent with Doctor” higher than “Accessibility and Convenience,” and female participants were significantly more satisfied than males. Satisfaction with health care delivery among all participants increased significantly comparing timepoints.

**Conclusions:**

Participants were highly satisfied with HCV telemedicine encounters equivalent to in-person encounters. Communication augments trust facilitating delivery of patient-centered care through telemedicine. Participants value empathy and trust with providers over accessibility and convenience. In summary, PWOUD are highly satisfied with the facilitated telemedicine model and value empathetic and trusting providers.

ClinicalTrials.gov Identifier: NCT02933970.

## Introduction

Access to satisfactory health care can be challenging particularly for vulnerable populations. Telemedicine, two-way interaction between a patient and a provider separated geographically, may circumvent these obstacles.^1,2^ Recent evidence-based systematic reviews have reported that telemedicine-based clinical outcomes are at least equivalent to or better than in-person care.^3–5^ Patient satisfaction with telemedicine, especially when targeted to vulnerable populations, including people with opioid use disorder (PWOUD), remains largely undefined.

According to the Institute of Medicine, high-quality care is safe, efficient, timely, patient centered, and equitable.^6^ For telemedicine to achieve this designation, especially when targeted to PWOUD, patient satisfaction, patient centeredness, and equitability must be prioritized. Specifically, how does substituting in-person interactions with telemedicine affect empathy conveyed during health care encounters? What attributes among the PWOUD population might improve satisfaction with telemedicine?^7^

PWOUD have the highest hepatitis C virus (HCV) incidence and prevalence.^8^ Referral to a liver specialist has been the conventional HCV management strategy. Due to stigma and other competing priorities, many HCV-infected PWOUD, however, elect not to pursue HCV treatment when referred.^9–13^ Consequently, PWOUD access to curative HCV therapy remains limited. Telemedicine integrated into the nonstigmatizing environment within opioid treatment programs (OTPs) has been shown to be a promising HCV treatment delivery modality.^4,14,15^ Furthermore, PWOUD appear to prefer the convenience and accessibility of telemedicine encounters compared with offsite referral. As PWOUD typically consider OTPs comfortable and familiar environments with reduced stigma compared to conventional health care delivery sites, these sentiments may translate into high satisfaction with telemedicine.^16,17^

We conducted a mixed-methods study to assess PWOUD satisfaction with health care delivery among individuals who had successfully completed HCV treatment, either through offsite referral to an HCV provider or through telemedicine encounters situated onsite in the OTP. We initially administered the Patient Satisfaction Questionnaire (PSQ)^18^ at two time points, and we subsequently conducted interviews to explore participants' experiences of facilitated telemedicine. The insights learned through this investigation may have broad applicability to achieving high satisfaction with telemedicine encounters targeted to vulnerable populations.

## Methods

### STUDY DESCRIPTION

All study participants included in this analysis are part of an ongoing stepped wedge cluster randomized controlled trial that is comparing the HCV cure rates among PWOUD treated through telemedicine conducted onsite in OTPs with offsite referral. The study was approved by the University at Buffalo Institutional Review Board (IRB) and the IRB at each study site. The analysis we conducted is “as treated,” meaning that the 344 participants had provided PSQ scores at both time points without encountering any missing values. PWOUD who obtained treatment for HCV infection either onsite in one of 12 participating OTPs in New York State or through offsite referral completed the PSQ at the initial and last provider encounters. All study participants had to be actively enrolled in one of the OTPs for at least 6 months before assessment of study eligibility and had to be HCV antibody and HCV RNA positive. Potential study participants were referred by OTP staff to study-supported case managers (CMs) who then conducted all screening activities.

All telemedicine encounters were facilitated by CMs and occurred entirely within the OTP. The CM situated the participant in the area designated for telemedicine and addressed all telemedicine-associated technical issues. We also sought to maximize telemedicine encounter quality using sponsor financial support to provide uniform wide-screen computers with high-quality cameras, microphones, and speakers that were distributed to all sites. As part of the study eligibility determination process, all potential participants underwent serological testing for HIV and hepatitis B virus (HBV). Any HIV- or HBV-infected participants were treated for HCV according to HCV treatment guidelines.^19^ Participants were treated with direct acting antivirals for 2–3 months followed by 3 months to assess for viral elimination. The telemedicine providers, who were gastroenterologists/hepatologists or advanced practice providers working under the direction of the hepatologist, directed the care of cirrhotic patients with local referrals for radiologic or endoscopic procedures as appropriate. For a complete description of the trial, please see Talal et al.^20^

At the initial study visit, participants provided information about demographics, living arrangements, comorbid conditions, and socioeconomic status. They also completed the Drug Abuse Screen Test (DAST-10)^21,22^ and National Institute on Drug Abuse Quick Screen^23^ to provide information on substance use history. The DAST was also administered at the last time point. We utilized a mixed-methods approach guided by the theory of pragmatism, which combines quantitative and qualitative approaches to analyze data.^24,25^ We used an Explanatory Sequential mixed-methods design,^25^ initially assessing participant's satisfaction with health care delivery by questionnaire and subsequently by interviewing PWOUD for enhanced understanding and context. Pragmatism, as an underlying theory for mixed-methods research, supports pluralism in research methodology.^26^

### PSQ ADMINISTRATION AND SCORING

We utilized the short-form PSQ (Modified PSQ-18) that is composed of 18 questions distributed into seven subscales^18^ (*Supplementary Table S1*). We modified the PSQ-18 for HCV care and subsequently piloted it with a racial/ethnicity and literacy level diverse population to ensure comprehension. The outcome corresponds to the score for each participant per time point and is calculated as the average of all questions answered out of 18 and subsequently rounded to the nearest integer (see *Section 1* in *Supplementary Data*). Overall and subscale PSQ outcome results are presented in *Supplementary Table S2*.

### MODELING

The patient satisfaction response scores, originally recorded on a 5-point scale, are modeled using a partial proportional odds model (see *Section 2* in *Supplementary Data*). We fit the cumulative model for ordinal data, using each participant's average score per time point as illustrated in *Supplementary Data*. Model covariates are presented in *Supplementary Table S3*. We included demographic covariates, such as race, ethnicity, gender, and age that have been shown to be important determinants of telemedicine encounter completion and satisfaction.^15^ We also assessed socioeconomic and health-related covariates we previously identified as promoting satisfaction with telemedicine among PWOUD.

Due to limited data on telemedicine satisfaction in PWOUD, we assume the effect of covariates time, arm, age, gender, highest level of education, combined monthly income, residence type, and comorbid conditions on PSQ scores as being the same across categories. In contrast, covariates race and ethnicity are not assumed to have the same effect across scoring categories. The Generalized Linear Mixed Models permit ordinal outcomes that are not normally distributed and account for repeated measurements.

### PARTICIPANT INTERVIEWS AND QUALITATIVE ANALYSIS

We used purposive sampling to obtain a representative sample of interviewees from the 238 telemedicine participants, consistent with the study design for hermeneutic studies.^27^ As our goal was to understand the experiences and to explicate common meanings of PWOUD undergoing HCV care through telemedicine integrated into an OTP, we interviewed participants who were referred by CMs, OTP staff, or members of the sites' patient advisory committees. After obtaining informed consent to conduct the interviews, we explored participants' experiences with HCV treatment through telemedicine using open-ended questions to maximize participant elaboration. We used the hermeneutic phenomenological research approach to understand patients' common meanings of HCV treatment integrated in an OTP (see *Supplementary Figure S1* and *Section 3* in *Supplementary Data*).^28–30^

### CONSTRUCTION OF WEIGHTS

We utilized NVivo (QSR International, Burlington, MA) to determine the frequency of specific code or word mention by participants and calculated the term frequency (tf), as well as the inverse document frequency (idf). The idf is the natural logarithm of the fraction of the number of documents (i.e., *N* = 25) over the number of documents containing the codes/words. We then calculate a normalized weight factor (WF) (tf − idf), which indicates the code/word's importance on a (0,1) scale as part of the interviews. In the case that there is more than one subtheme, the average WF is computed (see *Section 3* in *Supplementary Data*).

## Results

### POPULATION DESCRIPTION

We analyzed study participant responses to the PSQ at both time points (344 in total, 106 in the referral and 238 in the telemedicine arms). Sociodemographic characteristics are illustrated (*Tables 1* and *2*). The mean age is 48 ± 13 years; most participants are male (63.95%), Non-Hispanic (71.80%), and White (52.03%). Most participants use illicit drugs (61%) and reside in a private residence (85.76%). Approximately one third (38.08%) does not or is unsure whether they have a comorbid condition. Most participants (40.70%) had attended high school or obtained an equivalency degree, and one-third (35.47%) were in the highest category of monthly income.

**Table 1.** Study Demographics, Social Characteristics, and Substance Use Characteristics of All (344) Participants

VARIABLE	CATEGORY OR DESCRIPTIVE STATISTICS	NO. (%)^a^		
		REFERRAL (*N* = 106)	TELEMEDICINE (*N* = 238)	TOTAL (*N* = 344)
Age (at consent)	Mean ± SD	51.64 ± 12.01	46.36 ± 13.14	47.99 ± 13.02
Gender	Female	32 (30.19)	92 (38.66)	124 (36.05)
	Male	74 (69.81)	146 (61.34)	220 (63.95)
Hispanic or Latina	Yes	26 (24.53)	71 (29.83)	97 (28.20)
	No	80 (75.47)	167 (70.17)	247 (71.80)
Race	Black/African American	32 (30.19)	37 (15.55)	69 (20.06)
	Other (including American Indian/Alaska Native, Asian, Multiracial, Other)	25 (23.58)	71 (29.83)	96 (27.91)
	White	49 (46.23)	130 (54.62)	179 (52.03)
Comorbid condition	Anxiety and/or depression	26 (24.53)	82 (34.45)	108 (31.40)
	other comorbid conditions but not anxiety/depression	31 (29.25)	74 (31.09)	105 (30.52)
	No comorbid condition or unsure	49 (46.23)	82 (34.45)	131 (38.08)
Residence type	Other	12 (11.32)	37 (15.55)	49 (14.24)
	Private residence	94 (88.68)	201 (84.45)	295 (85.76)
Highest level of education	12th grade/completed high school or received GED/post high school training	44 (41.51)	96 (40.34)	140 (40.70)
	Some college/college graduate/postgraduate	28 (26.42)	55 (23.11)	83 (24.13)
	Less than or equal to 11th grade	34 (32.08)	87 (36.55)	121 (35.17)
Combined monthly income	High income ($1250 or more)	34 (32.08)	88 (36.97)	122 (35.47)
	Middle income ($834–$1249)	33 (31.13)	72 (30.25)	105 (30.52)
	Low income ($0–$833)	39 (36.79)	78 (32.77)	117 (34.01)
DAST-10^b^				
DAST-10 at	Mean ± SD	4.25 ± 3.18	4.73 ± 3.09	4.59 ± 3.12
Timepoint 1	Median; IQR	4.00; 6.00	5.00; 5.00	5.00; 5.00
DAST-10 at	Mean ± SD	3.07 ± 2.89	3.66 ± 2.88	3.47 ± 2.89
Timepoint 2	Median; IQR	2.00; 4.00	3.00; 5.00	3.00; 5.00

NIDA QUICK SCREEN				
IN THE PAST YEAR, HOW OFTEN HAVE YOU USED THE FOLLOWING?	ANSWERS PER QUESTION	NO. (%)^a^		
		REFERRAL (*N* = 106)	TELEMEDICINE (*N* = 238)	TOTAL (*N* = 344)
Alcohol: for men (women), 5 (4) or more drinks a day.	Never	83 (78.30)	185 (77.73)	268 (77.91)
	Once or twice	19 (17.92)	30 (12.61)	49 (14.24)
	Monthly	1 (0.94)	10 (4.20)	11 (3.20)
	Weekly	2 (1.89)	7 (2.94)	9 (2.62)
	Daily or almost daily	1 (0.94)	6 (2.52)	7 (2.03)
Tobacco products	Never	15 (14.15)	30 (12.61)	45 (13.08)
	Once or twice	3 (2.83)	1 (0.42)	4 (1.16)
	Monthly	2 (1.89)	1 (0.42)	3 (0.87)
	Weekly	1 (0.94)	4 (1.68)	5 (1.45)
	Daily or almost daily	85 (80.19)	202 (84.87)	287 (83.43)
Prescription drugs for nonmedical reasons	Never	79 (74.53)	166 (69.75)	245 (71.22)
	Once or twice	15 (14.15)	44 (18.49)	59 (17.15)
	Monthly	6 (5.66)	9 (3.78)	15 (4.36)
	Weekly	1 (0.94)	8 (3.36)	9 (2.62)
	Daily or almost daily	5 (4.72)	11 (4.62)	16 (4.65)
Illegal drugs	Never	48 (45.28)	86 (36.13)	134 (38.95)
	Once or twice	18 (16.98)	31 (13.03)	49 (14.24)
	Monthly	13 (12.26)	46 (19.33)	59 (17.15)
	Weekly	15 (14.15)	37 (15.55)	52 (15.12)
	Daily or almost daily	12 (11.32)	38 (15.97)	50 (14.53)

^a^ The percentages were calculated over the 344 participants.

^b^ The score variable for DAST-10 is calculated as the total number of “Yes” responses (which receive 1 point each), except for one question for which “No” receives 1 point. A score from 3 to 5 represents a moderate degree of problems related to drug abuse, and further investigation is suggested as outlined in references 19 to 21.

DAST, drug abuse screening test; GED, general educational development; IQR, interquartile range; NIDA, National Institute on Drug Abuse; SD, standard deviation.

**Table 2.** Study Demographics, Social Characteristics, and Substance Use Characteristics of the (25) Interviewees

DEMOGRAPHICS OF INTERVIEWED PARTICIPANTS		TOTAL TELEMEDICINE PARTICIPANTS	
VARIABLE	CATEGORY OR DESCRIPTIVE STATISTICS	NO. (%)^a^	
		TELEMEDICINE (*N* = 25)	TELEMEDICINE (*N* = 238)
Age (at consent)	Mean ± SD	46.8 ± 13.3	46.36 ± 13.14
Gender	Female	8 (32.00)	92 (38.66)
	Male	17 (68.00)	146 (61.34)
Hispanic Or Latina	Yes	6 (24.00)	71 (29.83)
	No	19 (76.00)	167 (70.17)
Race	Black/African American	6 (24.00)	37 (15.55)
	Other (including American Indian/Alaska Native, Asian, multiracial, other)	7 (28.00)	71 (29.83)
	White	12 (48.00)	130 (54.62)
Comorbid condition	Anxiety and/or depression	4 (16.00)	82 (34.45)
	other comorbid conditions but not anxiety/depression	9 (36.00)	74 (31.09)
	No comorbid condition or unsure	11 (44.00)	82 (34.45)
Residence Type	Other	5 (20.00)	37 (15.55)
	Private residence	20 (80.00)	201 (84.45)
Highest Level of Education	12th grade/completed high school or received GED/post high school training	11 (44.00)	96 (40.34)
	Some college/college graduate/post-graduate	7 (28.00)	55 (23.11)
	Less than or equal to 11th grade	7 (28.00)	87 (36.55)
Combined Monthly Income	High income ($1250 or more)	8 (32.00)	88 (36.97)
	Middle income ($834–$1249)	7 (28.00)	72 (30.25)
	Low income ($0–$833)	10 (40.00)	78 (32.77)
DAST-10^b^			
DAST-10 at	Mean ± SD	3.76 ± 3.36	4.73 ± 3.09
Timepoint 1	Median; IQR	3.00; 5.00	5.00; 5.00
DAST-10 at	Mean ± SD	3.12 ± 2.55	3.66 ± 2.88
Timepoint 2	Median; IQR	2.00; 5.00	3.00; 5.00

NIDA QUICK SCREEN			
IN THE PAST YEAR, HOW OFTEN HAVE YOU USED THE FOLLOWING?	ANSWERS PER QUESTION	NO. (%)	
Alcohol: For men (women), 5 (4) or more drinks a day	Never	19 (76.00)	185 (77.73)
	Once or twice	4 (16.00)	30 (12.61)
	Monthly	1 (4.00)	10 (4.20)
	Weekly	1 (4.00)	7 (2.94)
	Daily or almost daily	0 (0.00)	6 (2.52)
Tobacco Products	Never	1 (4.00)	30 (12.61)
	Once or twice	0 (0.00)	1 (0.42)
	Monthly	0 (0.00)	1 (0.42)
	Weekly	0 (0.00)	4 (1.68)
	Daily or almost daily	24 (96.00)	202 (84.87)
Prescription Drugs for Nonmedical Reasons	Never	19 (76.00)	166 (69.75)
	Once or twice	3 (12.00)	44 (18.49)
	Monthly	0 (0.00)	9 (3.78)
	Weekly	2 (8.00)	8 (3.36)
	Daily or almost daily	1 (4.00)	11 (4.62)
Illegal Drugs	Never	13 (52.00)	86 (36.13)
	Once or twice	3 (12.00)	31 (13.03)
	Monthly	4 (16.00)	46 (19.33)
	Weekly	1 (4.00)	37 (15.55)
	Daily or almost daily	4 (16.00)	38 (15.97)

^a^ The percentages were calculated over the 25 interviewees.

^b^ The score variable for DAST-10 is calculated as the total number of “Yes” responses (which receive 1 point each), except for one question for which “No” receives 1 point. A score from 3 to 5 represents a moderate degree of problems related to drug abuse, and further investigation is suggested as outlined in references 19 to 21.

DAST, drug abuse screening test; GED, general educational development; IQR, interquartile range; NIDA, National Institute on Drug Abuse; SD, standard deviation.

### HIGH SATISFACTION WITH TELEMEDICINE ENCOUNTERS, EQUIVALENT TO IN-PERSON ENCOUNTERS

Overall health care satisfaction was rated high (i.e., 96.2% [scores ≥4 at timepoint 1] and 96.5% [scores ≥4 at timepoint 2]) among all study participants (*Supplementary Table S2*). At the second timepoint, an ∼10% shift in scores occurred, an increase by one point (i.e., 4–5) in comparison with the initial timepoint. Less than 2% of patients were dissatisfied or highly dissatisfied (i.e., scored values 1 or 2) overall or to any of the subscales per timepoint.

### ATTRIBUTES OF TELEMEDICINE SATISFACTION FROM PARTICIPANT INTERVIEWS

We interviewed 25 telemedicine study participants to understand the factors, communication about the study, trust, and patient-centered care, that led to high satisfaction with telemedicine (*Table 3*). Through Communicating information promoting study enrollment and retention (Theme 1), participants discussed the importance of communication and transparency with OTP and study staff. “Every time I asked a question, they answered.” Participant's desire for HCV education and support enabled them to overcome skepticism and to accept HCV treatment and follow-up through telemedicine. “I know for myself that in the black community, we're skeptical about a lot of things medical, very skeptical.” Communication promotes Gaining trust in the OTP (Theme 2). Participants described meanings, such as the trust that emanates from the venue and the providers. Trust was able to mitigate anxiety toward telemedicine encounters and alleviate privacy, confidentiality, and security concerns. As one participant indicated, “The atmosphere in the clinic, they're very confidential.” Over time, participants became more comfortable with telemedicine.

**Table 3.** Evidence Supporting Participants' Experiences with Hepatitis C Virus-Related Health Care Delivery

THEME	MEANINGS	SUPPORTING QUOTE^a^
Theme 1: Communicating information promoting study enrollment, retention, and beyond	Valuing communication of medical information	“Every time I asked a question, they answered… Every time they gave me something, they described exactly what it was doing from beginning to end.”
	Becoming motivated to pursue HCV care	“When they show you a schematic of your liver and [my] thoughts get to the scary point, to cirrhosis level… there is no coming back from that.”
	Supporting HCV treatment pursuit though staff communication	“They introduced me to the study and the follow up for that study help[ed] me with insurance and everything …. The staff was really professional, and I got to the hepatitis study thanks to the staff.”
	Accepting HCV treatment procedures and protocols	“I'm all for it. That giving up blood and everything, to me, was just part of it. That's a small price to pay.”
	Being skeptical and lacking computer access	“I know for myself that in the black community, we're skeptical about a lot of things medical, very skeptical. So, it has to be a way, and also there's a lot of people in the black community that don't have access to computers and stuff.”
	Recommending improvements: Enhanced information access	“If you just came up with frequently asked questions…basic things, problems that you would probably run across, if you accidentally skip a day, what should you do, or what to take the medication with, water?…that would be helpful.”
	Promoting facilitated telemedicine through outreach and advocacy	“I think it needs advertisement to let people know.” “Now, if there were a way they could have some apps on the phone and maybe a 15-second commercial, that would make a lot more people aware.”
	Suggesting practical improvements: provider access, frequently asked questions sheet, scheduling assistance	“Maybe having some kind of direct contact with the doctor… because there were a few times that I had a few questions about the medication…. missed a day…what to do? I ended up calling the clinic, so maybe if you had all that information ahead of time, who you could talk to if you have a problem [it might be helpful].” “Once they gave people different timing to come in, so everyone has their own time to come in [is helpful].”
Theme 2: Gaining trust in the OTP to participate in patient-centered telemedicine encounters	Dissipating apprehension toward telemedicine	“I was a little apprehensive at first, but …everything is explained…it was informative.”
	Valuing telemedicine encounter in familiar venue	“A lot of addicts have issues with anxiety…., so I would probably just say, telemedicine is a much easier way to communicate without having to feel afraid of saying what you really want to say.” “You're more comfortable in your home… you're more at ease, and if I have questions for the doctor, you're not going to forget. When you're in the doctor's office, you're more nervous, and I tend to forget it.”
	Trusting community	“Really understood me and adapted to me personally in my needs.” “For me, they've always been very nice, friendly and outgoing…very tight.”
	Promoting trust in study and HCV treatment through patient-staff relationships	“I was getting it here [OTP clinic], it helped me to finish it, and I was being monitored every day… They asked me how I felt, ‘are you all right, experiencing anything’, and they reassured me.” “Everybody here is very respectful, very good people…. not only him [study doctor], the PA… talked to me a lot about it [HCV treatment] … she was there [to facilitate the TM].”
	Appreciating privacy, confidentiality, and security	“The atmosphere in the clinic, they're very confidential. They didn't let anyone know that I was involved in the Hep C program…. they were professional…. I like that it was confidential because I prefer no one know that this is what I'm going through.”
Theme 3: Realizing advantages of patient-centered, virtually integrated HCV treatment	Saving time and cost in a familiar venue	“I would absolutely recommend it, especially if … a lot of addicts can be like me, where they don't want to go to hospitals, they don't want to sit in doctors' offices. And this might make it easier for them to get treatment.” “I'm looking at it from the terms of my experience as a black person that a lot of people don't have jobs that they can just step away from to attend a doctor's appointment. You save your doctor's appointments for when you need to go to the doctor. But if there's a person that just needs to get his high blood pressure medicine, it's fine, it's perfect [telemedicine].” “It's very easy and very convenient because I don't have to go nowhere… All the appointments and communications with the doctors were done through the computer, it really saves you time.”
	Overcoming treatment obstacles and practical approaches	“The experience for telemedicine was convenient, because it was at the clinic, I didn't have to travel somewhere else.” “[integrated telemedicine] it's a really good idea, because I'm one who puts things off every day, so if I come into the program and, if I have an opportunity to do it over the computer, I mean let me do it [HCV treatment] here.” “it's [telemedicine] almost better than seeing the doctor once in a blue moon because I'm going to my clinic frequently, so, if a problem arises… I can confront it fast, it wasn't frightening.”
	Appreciating facilitated virtual encounters	**“**I think the telemedicine part was great… And having the lady [clinical liaison] with me…if I didn't understand what the doctor said, she helped clarify it.” “It felt a little strange at first not having the [doctor] person in the room, but they were straight to the point, they asked me questions, and to tell you the truth, it felt like the person was there.”
	Valuing HCV cure on OUD recovery	“At the time… that I came to get my methadone, which was definitely a lifeline at that point for me. It was definitely great that I could come here and do the telemedicine thing, which helped save my life when it comes to hepatitis C.” “Well, it certainly unburdened me by knowing that I had [been cured] of Hep C [be]cause I've had numerous friends die from liver failure from Hep C.”
	Evaluating usefulness of telemedicine	“My experience was good. I don't think telemedicine is for everybody or for everything, like if you hurt your arm or have a knee or bone problem, telemedicine is not going to help you.” “Maybe some people are more comfortable meeting in person to talk to the doctor.”
		“#1, you get your questions answered quick. #2, if there's a mistake, you don't have to wait for another 30 days for another appointment. #3, if there is anything that you need, it is at the tip of your phone. And that's a good thing, gives you open access, it doesn't have to be your doctor, as long as the people that know your case, they can pull your medical history up right here, and it's not invading your privacy.”
	Cost of HCV cure	**“**I heard it [costs] so much money … there are people probably that really would love to have it, but they can't afford it.”

^a^ All quotes presented are from different participants.

HCV, hepatitis C virus; OTP, opioid treatment program; OUD, opioid use disorder; PA, physician's assistant; TM, telemedicine.

Over time participants described Realizing advantages of patient-centered HCV care (Theme 3). Participants recognized the tangible advantages of ready access to HCV providers. They also recognized the convenience of collocated HCV and opioid use disorder (OUD) treatment. Individuals with questionable adherence due to active addiction especially value integrated HCV and OUD care. “I would absolutely recommend it, especially if … a lot of addicts can be like me, where they don't want to go to hospitals, they don't want to sit in doctors' offices.” Participants also appreciated how an HCV cure is integral to substance use recovery.

### ATTRIBUTES OF HEALTH CARE DELIVERY SATISFACTION AT THE ENCOUNTER LEVEL

Participant interviews provided insight into attributes that increased telemedicine satisfaction over the course of the entire study. We next sought to investigate the specific attributes associated with health care delivery satisfaction at the encounter level. The evaluation of the overall PSQ and subscale scores revealed that the three most frequently mentioned codes and weighting factor were “Time Spent with Doctor,” “General Satisfaction,” and “Interpersonal Manner.” Less frequently mentioned were “Technical Quality” and “Accessibility and Convenience” (*Table 4*). These results suggest that study participants valued trust and empathy over technical aspects or accessibility and convenience.

**Table 4.** Correspondence Between the Patient Satisfaction Questionnaire Responses and Coded Themes and Words

PSQ-SUBSCALES	CODED THEMES	PERCENTAGE OF CODE MENTIONING^a^	AVERAGE OF PERCENTAGES	NORMALIZED WF: MEAN (SD)	OVERALL AVERAGE^b^
Accessibility and convenience	Convenience	20	24	0.082 (0.186)	0.091
	Privacy	28		0.099 (0.177)	
Technical quality	Recommending treatment	56	56	0.135 (0.151)	0.135
Interpersonal manner	Person-centered care	72	72	0.155 (0.129)	0.155
General satisfaction	Gratitude	84	88	0.168 (0.110)	0.177
	HCV treatment as cure^**c**^	92		0.186 (0.074)	
Time spent with doctor	TM as facilitated HCV Treatment	88	88	0.181 (0.087)	0.181
Communication^d^	—	—	—	—	—
Financial aspects^d^	Study inclusion/exclusion criteria	—	—	—	—

^a^ Percentages are calculated as the number of the reference frequency out of 25 interviewed participants.

^b^ Overall average refers to average of normalized WF means.

^c^ Communication about the entire spectrum of participation (i.e., from research engagement through post-treatment follow-up) as opposed to specifically referring to telemedicine encounters.

^d^ Dashes indicate that there were no mentions during telemedicine encounters.

HCV, hepatitis C virus; PSQ, patient satisfaction questionnaire; TM, telemedicine; WF, weight factor.

Note: Codes and themes are mapped into the different PSQ subscales. The frequency (counts) of codes is used in the construction of the normalized score, indicating the weight participants assigned to different PSQ subscales.

### CHANGES IN ATTRIBUTES OF SATISFACTION WITH HEALTH CARE DELIVERY OVER TIME

When evaluating the changes in individual PSQ subscales comparing timepoints 1 and 2, we noted substantial improvements at timepoint 2 in “General Satisfaction,” “Time Spent with Doctor,” and “Accessibility and Convenience” (see *Supplementary Figure S2* and *Section 4* in *Supplementary Data*).

When adjusting for covariates, we observed that overall satisfaction improved significantly (*p* = 0.0015, 95% confidence interval [CI]: −5.2618 to −1.2488) comparing the last and the initial timepoints (*Table 5*). The time coefficient is −0.7155 indicating that participants at the second timepoint have a higher probability of assigning scores in the higher patient satisfaction categories in comparison to the first timepoint.

**Table 5.** Participant Satisfaction Scores Modeled as a Function of Time and Participants' Characteristics (Significant Results Are Only Presented)

MODELING RESULTS AMONG ALL PARTICIPANTS (#344)							
PARAMETER		ESTIMATE	STANDARD ERROR	t	PR > |t|	LOWER LIMIT OF 95% CI	UPPER LIMIT OF 95% CI
α_1_	Intercept 1 (scores 1–3 vs. scores 4 or 5)	−3.2553	1.0201	−3.19	0.0015	−5.2618	−1.2488
α_2_	Intercept 2 (scores 1–3 or 4 vs. score 5)	2.0105	0.9244	2.17	0.0303	0.1922	3.8287
β_2_	Timepoint (0 = 1st time point; 1 = 2nd time point)	−0.7115	0.2082	−3.42	0.0007	−1.1210	−0.3021
β_3_	Gender (0 = male; 1 = female)	−0.7376	0.3520	−2.10	0.0368	−1.4299	−0.0454
γ*l*[*i*	Variance 2 (participants nested within clinic)	4.6533	1.1851	3.93	0.0001	2.3224	6.9843

CI, confidence interval.

The level of the categorical variable, which takes value equal to 0, corresponds to the reference level.

Significant differences between males and females were observed (*p* = 0.0368, 95% CI: −1.4299 to −0.0454). The coefficient of gender is −0.7376 indicating that female participants have a higher probability of assigning scores in the higher patient satisfaction categories in comparison to male participants. There are also two intercept terms that correspond to the two cumulative logits defined on the score categories (scores 1–3 vs. scores 4 or 5 and scores 1–3 or 4 vs. score 5, respectively) with respective *p*-values 0.0015 and 0.0303 and respective estimates −3.2553 and 2.0105. These results indicate that the participants are more likely to assign higher scores (4 or 5) than lower scores (1–3) and less likely to assign the highest score (5) compared to the other scores (1–3 or 4). The nested patient level random effect is significant (*p* < 0.05), and the intraclass correlation coefficient is 0.5858. Thus, individual satisfaction scores vary across study sites, indicating site-to-site differences.

### PARTICIPANT SUGGESTIONS REGARDING IMPROVEMENTS IN TELEMEDICINE DELIVERY

Some participants recommended publicity to promote participation in telemedicine. “I think it needs advertisement to let people know.” Additional recommendations include provider contact information and education specifically targeted to individuals skeptical about medical technology (Theme 1). Furthermore, OTP and study staff played critical roles in initial engagement and retention in HCV treatment. “I was getting it [HCV treatment] here [OTP clinic]; it helped me to finish it” (Theme 2).

## Discussion

Participants in our investigation were equally satisfied with the facilitated telemedicine model and referral for HCV management. Based upon PSQ scores and participant interviews, we observed that satisfaction with health care delivery increased over time among telemedicine and referral participants. Specific attributes that improved PWOUD satisfaction with telemedicine were communication and education about the study, HCV, and telemedicine that promoted study participation and retention.^31,32^ Study-supported CMs were essential to facilitate addressing participants' competing priorities, assuaging their concerns, and answering their questions, all of which promoted satisfaction with telemedicine. Participants indicated that communication promotes trust in the OTP and by extension to the telemedicine encounters and providers. They further explained that trust in telemedicine as a health care delivery modality potentiates the provision of patient-centered HCV care.

Substitution of in-person encounters with telemedicine had minimal effect on empathy. This observation is based upon scores on the two relevant PSQ subscales, time spent with the doctor and the interpersonal manner. Females were significantly more satisfied with health care delivery than males. We understood that situating telemedicine encounters in the OTP promotes participant confidence in the security and confidentiality of the health care delivery modality. The combination of a trusting environment and an empathetic provider appears to promote telemedicine acceptance by PWOUD.

In our study, health care delivery through telemedicine adds value without compromising quality, as others have recently recommended.^33^ OTP clinical staff were available to review the patient's history, perform physical examinations, and answer questions. These actions reinforced connectivity with the telemedicine provider.^34^ Onsite phlebotomy facilitated data acquisition, a necessity since PWOUD rarely adhere to offsite laboratory referral. HCV treatment through telemedicine also increased visit adherence compared to usual care consistent with a recent study that reported 50% fewer “no shows” for telemedicine patients compared with in-person evaluations.^35^ The facilitated telemedicine model also increases value through simultaneously treating OUD and HCV by dispensing HCV medications with methadone. Contemporaneous HCV and OUD treatment has recently been shown to increase medication adherence, retention-in-care, and treatment effectiveness.^36–38^ The cumulative effect of these interventions is to increase satisfaction with telemedicine.

Telemedicine satisfaction and accessibility requires entry points that are safe, equitable, and patient centered.^33^ Our facilitated telemedicine model appears to decrease health care disparities as others have suggested^39^ and consistent with data from a recent study among persons experiencing homelessness.^40^ Another recent study illustrated high telehealth satisfaction among rural residents along the U.S.-Mexican border,^41^ and it enabled substance users to receive primary care during the COVID-19 pandemic.^42^

Colocating all telemedicine encounters in OTPs ensured adequate broadband strength and leveraged their familiar and destigmatizing environments. Frequent in-person attendance requirements for methadone treatment offer communication opportunities and promote encounter and medication adherence. We learned that explanation of study procedures and security and confidentiality safeguards promoted and reinforced comfort in digital technology, consistent with American College of Physicians' guidelines.^43^ Furthermore, participants indicated that education delivered by CM in a cultural- and literacy-appropriate manner can mitigate skepticism toward HCV and telemedicine as recommended by others.^44^ Our results are also consistent with a recent study that showed that telehealth familiarity can increase telemedicine completion rates.^32^ As the deployment and appropriate operation of telemedicine equipment to all individuals may be infeasible, additional research is needed to evaluate methods to utilize telemedicine creatively to decrease health care disparities. A facilitated telemedicine approach may be helpful in certain situations.

The use of mixed-methods methodology is a study strength. Patient interviews provided contextual understanding of the facilitated telemedicine experience, in which participants felt comfortable in the familiar OTP setting. Participant response weighting strengthened and quantified the importance of identified themes and codes. Participants noted that they felt connected to the telemedicine provider, and they valued behaviors designed to express empathy as recommended by others.^45^ In terms of limitations, we only measured patient satisfaction at two time points, we interviewed only telemedicine participants, and we had unequal numbers of telemedicine and usual care participants. Furthermore, additional research should assess the generalizability of the facilitated telemedicine model to other venues particularly those outside of New York State. For example, Medicare now reimburses providers for telemedicine examinations conducted in people's homes,^46^ as has been suggested for OUD treatment.^47^ We also noted site-to-site differences in participant satisfaction through modeling, and ongoing work is investigating the reasons for the site-specific differences.

## Conclusions

PWOUD satisfaction with telemedicine is equivalent to in-person care when delivered from destigmatized familiar sites by empathetic providers. Our facilitated telemedicine model using familiar staff as facilitators augments quality, adds value, and achieves high patient satisfaction. Participants experienced provider empathy virtually and developed trust over time. We also leveraged the accessibility and convenience of the familiar and comfortable OTP environment. Our findings of high satisfaction with telemedicine health care delivery are consistent with others who report high satisfaction with video visits across a variety of gastroenterology conditions not necessarily targeted to vulnerable populations.^32,48,49^ Future work should investigate if the model is generalizable to other venues and situations where telemedicine can deliver highly satisfactory health care, which simultaneously augments quality and adds value.

## Supplementary Material

Supplemental data
