# Supplementary material for: High Satisfaction with Patient-Centered Telemedicine for Hepatitis C Virus Delivered to Substance Users: A Mixed-Methods Study
Source: Telemed J E Health. 2023 Mar 10;29(3):395–407. doi: 10.1089/tmj.2022.0189 (PMC10024261; doi:10.1089/tmj.2022.0189)
Supplement: Supplemental data [file Supp_Data.docx]

**Supplementary material**

**High Satisfaction with Patient-Centered Telemedicine for Hepatitis C Virus Delivered to Substance Users: A Mixed-Methods Study**

The supplementary material contains the following items:

1. Supplementary Table S1
2. Supplementary Table S2
3. Supplementary Table S3
4. Supplementary Figure S1
5. Supplementary Figure S2
6. Supplementary Figure S3
7. **Description of Patient Satisfaction Questionnaire (PSQ) and its Subscales**

PSQ administration: We utilized the short-form PSQ (Modified PSQ-18) [1]. The instrument is comprised of 18 questions which fall into 7 subscales describing various dimensions of the medical care process: General Satisfaction (GS), Technical Quality (TQ), Interpersonal Manner (IM), Communication (Com), Financial Aspects (FA), Time Spent with Doctor (TSWD), Accessibility and Convenience (AC). The subscales have acceptable internal consistency and reliability. Each subscale is composed of an equal number of “positively- and negatively- worded items” (eTable 1). The responses are given on a 5-point Likert scale ranging from “1=Strongly Agree” to “5=Strongly Disagree”. We modified the PSQ-18 for hepatitis C virus (HCV) care and subsequently pilot tested it with a racial/ethnic and literacy level diverse population to ensure comprehension. We administered the PSQ-18 after completion of the first HCV provider visit (i.e., baseline) and at the sustained virological response (SVR) time point.

Score calculation: A score variable was calculated as follows: for questions 4, 7, 9, 10, 12, 13, 14, 16, 17 with negatively-worded items, the scored value is equivalent to the value of the original response; while for questions 1, 2, 3, 5, 6, 8, 11, 15, 18 with positively-worded items, the reversed value of the original response is used for providing the scored value. Thus, the higher the scored value, the higher the satisfaction. We calculated the score per participant, per time point, as the average of the scored values of all questions answered out of 18, and subsequently rounded the average score to the nearest integer. If the decimal part is less than 5, then the score is rounded down; otherwise, it is rounded up. We calculated the number of participants who provided a specific score along with the corresponding percentages, per time point. The results are presented in eTable 2.

1. **Modeling**

Outcome variable: The original outcome, measured on ordinal scale, was transformed to variable $Y$ which takes values 1, 2 or 3 representing the three average score categories: 1-3, 4, and 5. For example, $Y=1$ means that the participant has an average score of 1-3, $Y=2$ means that the participant has an average score of 4, and $Y=3$ means that the participant has an average score of 5. To avoid convergence issues that may be present because of the sparseness of data for scores 1-3, the aforementioned concatenation of categories of the outcome was necessary [2, 3].

Model fit for both arms: We fit the partial proportional odds cumulative logit model for ordinal multinomial data with participant random effects. eTable 3 presents the model covariates. Details on the treatment of individual covariates are given below. Score tests for the proportional odds assumptions were performed to investigate which covariates are proportional and which are not. The random intercepts in our model correspond to the participant-level random effect to account for the variability in the outcome among different participants and different participants nested within clinics, thereby accounting for clustering in the data. A total of 344 participants, who answered the PSQ at both time points (complete case analysis), are used to fit this model. The model used captures the information included in the data, allows us to adjust all covariates, and provides reasonable parameter explanation.

The outcome variable is $\log\left\{ \frac{r_{j}\left( x \right)}{1-r_{j}\left( x \right)} \right\}$, where $r_{j}\left( x \right)=\Pr(Y\leq j|x)$ is the cumulative probability up to and including category $j$, $j=1,2,\ldots J-1$ with $J$ indicating the number of categories. In our case, $J=3$, $Y\leq j$ is the event of falling into or below category $j$ ($j=1$ means ordinal score 1-3, $j=2$ means ordinal score 4). The bold-faced betas represent vectors and refer to categorical variables with more than two levels.

Model assumptions: The partial proportional odds model assumes that the effects of the covariates associated with Race and Ethnicity are not the same for all categories on the log odds scale, while the remaining covariates are assumed to have the same effect on all categories of the log odds scale, assumption that was justified by the score test. In addition, given the lack of data regarding the satisfaction with healthcare delivery, and particularly via telemedicine, we chose to examine the potential effects of race and ethnicity covariates, thereby forgoing the assumption of proportionality. The distributions of the random effects are normal with zero means and respective variances $\sigma_{\gamma_{l}}^{2}$, $\sigma_{\gamma_{l[i]}}^{2}$ [4, 5].

To fit this model, we used SAS 9.4, PROC NLMIXED procedure with general distribution, log-likelihood function and adaptive Gauss-Hermite quadrature method. The optimization and the integration technique applied were the Dual Quasi-Newton and the Adaptive Gaussian Quadrature, respectively. We chose the values of the initial parameters to be equal to the ones of the proportional odds model with a nested random effect that was fitted with PROC GLIMMIX and the pseudo-likelihood estimation method.

We also calculated the intraclass correlation (ICC) using the formula: $ICC=\sigma_{\gamma}^{2}/(\sigma_{\gamma}^{2}+\sigma^{2})$, where $\sigma_{\gamma}^{2}$ is the variance of the random effect and $\sigma^{2}$ is the residual variance, which equals the variance of the standard logistic, i.e., $\sigma^{2}=\pi^{2}/3$ [6].

Model covariates: eTable 3 presents the model’s covariates. The reference level is selected as the most normative group for the population under study [7]. For example, we used the middle income as the reference level in combined monthly income because it is within the limits of the Social Security Disability Income levels [8].

1. **Qualitative Analysis**

Recruitment of interview participants: Once we obtained the institutional review boards’ approvals, study case managers invited study participants to be interviewed. We used purposive sampling, consistent with hermeneutic study design [9]. In our case, the goal of hermeneutic analysis is to understand the experiences and to explicate common meanings of PWOUD undergoing onsite HCV care via telemedicine integrated in the OTP. Thus, we sought to interview telemedicine participants who would be willing to share their experiences. Participants in the parent study who obtained an HCV cure were referred by study staff or by members of the patient advisory committee at each study site. Table 2 describes the characteristics of this sample vis-a-vie the population of 238 telemedicine participants. We did not observe any significant differences between the population of 238 telemedicine participants and the 25 interviewees, so the subsample closely aligns with the overall population.

Participant interviews: To ensure adequate interview representation from each site, we invited ~3 participants per site. We interviewed 25 telemedicine study participants corresponding to a ~10% sample of the telemedicine participants. Participants were individually interviewed by one of two study staff members. Interviews were recorded and transcribed. We then deidentified and verified all transcriptions against interview recordings. We used the confirmed, deidentified text version of the interview as raw data for qualitative analysis utilizing hermeneutic phenomenology [9]. We explored participants’ experiences with HCV treatment and telemedicine for further elucidation of the PSQ subscales responses. Interview questions were open-ended to maximize participant elaboration.

Qualitative analysis: Hermeneutic phenomenology seeks to understand situations and interactions as they were experienced, within a context of time, place, and situational influences [10-12]. Interpretation of meanings is revealed through the spoken language of experiences, as captured in the interviews. Interpretation is iterative, circular, reflective and reflexive, dynamically engaging in thinking that arrives at a current understanding of human situations as they are experienced [10, 12]. Reflection involves making explicit the interpreters’ presuppositions, creating an engaged openness and closeness with the text to understand the human situation as lived, and it involved reflections by all members of the interpretive team [11]. An algorithm depicting the qualitative analytical methods employed is illustrated in eFigure 1.

The interview transcripts served as the basis for interpretation or a rendering of the interpretation or “fusion of horizons” [11]. The three-member analysis team included the study principal investigator (AHT), an expert in hermeneutic qualitative analysis (SSD); and the overall project manager (UJ). SSD guided the analysis. The team interpreted the texts in a reflective process that followed iterative steps [9, 13]. The related codes (i.e., texts linked by a common theme) and themes were examined by the team for coherence and comprehensiveness [14].

Rigor was maintained throughout the analysis phase in accordance with DeWitt and Ploeg’s framework of balanced integration, openness, concreteness, resonance, and actualization [15]. In the interpretation of study findings, we achieved balanced integration by balancing the voice of the participants and the researchers’ interpretations. Study results, as explicated in codes and themes, use both verbatim excerpts and interpretations.

Term frequency determination: We next sought to associate codes and words derived from the interviews with patient satisfaction among telemedicine participants by calculating appropriate text mining metrics. Specifically, we assessed the relatedness between the code frequency and satisfaction as expressed through PSQ subscales as well as that between words with covariates significantly affecting participant satisfaction (see Table 4).

We calculated text mining metrics [16]. In particular, we calculated *the term* $(t)$ *frequency* that is the number of times that a term is mentioned in a document, and defined $TF\left( t,d_{i} \right)=df(t,d_{i})$, where $df\left( t,d_{i} \right)$ is the number of times $t$ appears in document $d_{i},i=1,2,\ldots,N=25.$ The *inverse document frequency* ($IDF$) is defined as $IDF\left( term \right)=ln\left\{ \frac{1+N}{1+TF\left( t,d_{i} \right)} \right\}+1$, and the $TF-IDF(t,d_{i})=TF\left( t,d_{i} \right)\times IDF\left( t \right)$. Subsequently, we calculated the weights attributed to codes and significant words by normalizing the scores on a $(0,1)$ scale using the formula Weight factor (WF) $WF=Normalized\_TF-IDF(t,d_{i})=\left[ TF-IDF\left( t,d_{i} \right) \right]/z$, where $z=\left\{ \sum_{i=1}^{N} \left[ TF-IDF(t,d_{i}) \right]^{2} \right\}^{1/2}$. The results are presented in Table 4, and they indicate the importance placed by participants on the different aspects of medical care delivery.

In addition to these metrics, we calculated the percentages of the answers provided, as not all of the 25 interviewees had mentioned a specific code or word in their interviews (in these cases $TF\left( t,d_{i} \right)=df\left( t,d_{i} \right)=0$). For example, in the PSQ subscale, TQ, the corresponding percentage is 56%, as only 14 patrticipants out of 25 interviewees mentioned the associated code “Recommending Treatment”. In the cases in which more than one code/word was used, the average of the sub-percentages is also presented (see Table 4).

1. **Results and Explanations**

Graphical analysis of PSQ scores: eFigure 2 shows barplots of mean PSQ score differences (T2-T1) between the two time points for all 7 subscales of healthcare delivery, as well as the overall score. The figure depicts participant’s difference in scores among all ($N=344$) and telemedicine ($N=238$) participants. eFigure 2 indicates that there is higher satisfaction with healthcare delivery at the second time point (all differences are positive). PSQ subscales GS, TSWD and AC depict larger differences in scores (T2-T1). We also illustrate the scores at the two time points per subscale and per arm (see eFigure 3). eFigure 3 shows that the same three PSQ subscales (GS, TSWD and AC) differ between the two time points within each arm.

**Supplementary Table S1:** Patient Satisfaction Questionnaire Subscales

| **PSQ-18 Subscale** | **PSQ-18 Sample Question [1]** | **Sample HCV Question** |
| --- | --- | --- |
| 1. General Satisfaction | 3. The medical care I have been receiving is just about perfect. | 3. The Hep C care I have been receiving is just about perfect. |
|  | 17. I am dissatisfied with some things about the medical care I receive. | 17. I am dissatisfied with some things about the Hep C care I receive. |
| 2. Technical Quality | 2. I think my doctor’s office has everything needed to provide complete care. | 2. I think the facility I go to for Hep C care has everything needed to provide complete medical care. |
|  | 4. Sometimes doctors make me wonder if their diagnosis is correct. | 4. Sometimes my Hep C doctor makes me wonder if their diagnosis is correct. |
|  | 6. When I go for medical care, they are careful to check everything when treating and examining me. | 6. When I go for Hep C care, they are careful to check everything when treating and examining me. |
|  | 14. I have some doubts about the ability of the doctors who treat me. | 14. I have some doubts about the ability of the Hep C doctor who treats me. |
| 3. Interpersonal Manner | 10. Doctors act too businesslike and impersonal toward me. | 10. My Hep C doctor acts too businesslike and impersonal toward me. |
|  | 11. My doctors treat me in a very friendly and courteous manner. | 11. My Hep C doctor treats me in a very friendly and courteous manner. |
| 4. Communication | 1. Doctors are good about explaining the reason for medical tests. | 1. My Hep C doctor is good about explaining the reason for medical tests. |
|  | 13. Doctors sometimes ignore what I tell them. | 13. My Hep C doctor sometimes ignores what I tell them. |
| 5. Financial Aspects | 5. I feel confident that I can get the medical care I need without being set back financially. | 5. I feel confident that I can get the Hep C care I need without being set back financially. |
|  | 7. I have to pay for more of my medical care than I can afford. | 7. I have to pay for more of my Hep C care than I can afford. |
| 6. Time Spent with Doctor | 12. Those who provide my medical care sometimes hurry too much when they treat me. | 12. Those who provide my Hep C care sometimes hurry too much when they treat me. |
|  | 15. Doctors usually spend plenty of time with me. | 15. My Hep C doctor usually spends plenty of time with me. |
| 7. Accessibility and Convenience | 8. I have easy access to the medical specialists I need. | 8. I have easy access to the Hep C doctor I need. |
|  | 9. Where I get medical care, people have to wait too long for emergency treatment. | 9. Where I get my Hep C care, people have to wait too long for emergency treatment. |
|  | 16. I find it hard to get an appointment for medical care right away. | 16. I find it hard to get an appointment for Hep C care right away. |
|  | 18. I am able to get medical care whenever I need it. | 18. I am able to get Hep C care whenever I need it. |

Abbreviations: Hep c, hepatitis C virus.

**Supplementary Table S2:** Patient Satisfaction Questionnaire: Overall and Sub-scale Scores per Time Point

| **Results among all participants (#344)** | | | | | | |
| --- | --- | --- | --- | --- | --- | --- |
| **Mean Scores ^a^** | | **No. (%)^b^** | | | | |
|  |  | **1** | **2** | **3** | **4** | **5** |
| **Overall** | **T1** | 0 (0.00) | 1 (0.29) | 12 (3.49) | 215 (62.50) | 116 (33.72) |
|  | **T2** | 0 (0.00) | 0 (0.00) | 12 (3.49) | 184 (53.49) | 148 (43.02) |
| **General Satisfaction (GS)** | **T1_GS** | 0 (0.00) | 6 (1.74) | 17 (4.94) | 165 (47.97) | 156 (45.35) |
|  | **T2_GS** | 1 (0.29) | 3 (0.87) | 15 (4.36) | 136 (39.54) | 189 (54.94) |
| **Technical Quality (TQ)** | **T1_TQ** | 0 (0.00) | 0 (0.00) | 7 (2.04) | 190 (55.23) | 147 (42.73) |
|  | **T2_TQ** | 0 (0.00) | 0 (0.00) | 8 (2.33) | 164 (47.67) | 172 (50.00) |
| **Interpersonal Manner (IM)** | **T1_IM** | 0 (0.00) | 2 (0.58) | 12 (3.49) | 155 (45.06) | 175 (50.87) |
|  | **T2_IM** | 0 (0.00) | 2 (0.58) | 12 (3.49) | 133 (38.66) | 197 (57.27) |
| **Communication (Com)** | **T1_Com** | 0 (0.00) | 1 (0.58) | 5 (3.49) | 136 (45.06) | 202 (50.87) |
|  | **T2_Com** | 0 (0.00) | 1 (0.29) | 8 (2.33) | 116 (33.72) | 219 (63.66) |
| **Financial Aspects (FA)** | **T1_FA** | 0 (0.00) | 1 (0.29) | 15 (4.36) | 143 (41.57) | 185 (53.78) |
|  | **T2_FA** | 1 (0.29) | 0 (0.00) | 15 (4.36) | 129 (37.50) | 199 (57.85) |
| **Time Spent with the Doctor (TSWD)** | **T1_TSWD** | 0 (0.00) | 3 (0.87) | 25 (7.27) | 181 (52.62) | 135 (39.24) |
|  | **T2_TSWD** | 0 (0.00) | 3 (0.87) | 13 (3.78) | 164 (47.675) | 164 (47.675) |
| **Accessibility and Convenience (AC)** | **T1_AC** | 1 (0.29) | 4 (1.16) | 25 (7.27) | 226 (65.70) | 88 (25.58) |
|  | **T2_AC** | 1 (0.29) | 1 (0.29) | 23 (6.69) | 184 (53.49) | 135 (39.24) |
| **Results among telemedicine participants (#238)** | | | | | | |
| **Mean Scores ^a^** | | **No. (%)^c^** | | | | |
|  |  | **1** | **2** | **3** | **4** | **5** |
| **Overall** | **T1** | 0 (0.00) | 0 (0.00) | 4 (1.68) | 149 (62.61) | 85 (35.71) |
|  | **T2** | 0 (0.00) | 0 (0.00) | 3 (1.26) | 129 (54.20) | 106 (44.54) |
| **General Satisfaction (GS)** | **T1_GS** | 0 (0.00) | 0 (0.00) | 6 (2.52) | 118 (49.58) | 114 (47.90) |
|  | **T2_GS** | 0 (0.00) | 0 (0.00) | 6 (2.52) | 96 (40.34) | 136 (57.14) |
| **Technical Quality (TQ)** | **T1_TQ** | 0 (0.00) | 0 (0.00) | 3 (1.26) | 130 (54.62) | 105 (44.12) |
|  | **T2_TQ** | 0 (0.00) | 0 (0.00) | 4 (1.68) | 112 (47.06) | 122 (51.26) |
| **Interpersonal Manner (IM)** | **T1_IM** | 0 (0.00) | 0 (0.00) | 9 (3.78) | 109 (45.80) | 120 (50.42) |
|  | **T2_IM** | 0 (0.00) | 0 (0.00) | 8 (3.36) | 91 (38.24) | 139 (58.40) |
| **Communication (Com)** | **T1_Com** | 0 (0.00) | 0 (0.00) | 2 (0.84) | 95 (39.92) | 141 (59.24) |
|  | **T2_Com** | 0 (0.00) | 1 (0.42) | 2 (0.84) | 80 (33.61) | 155 (65.13) |
| **Financial Aspects (FA)** | **T1_FA** | 0 (0.00) | 0 (0.00) | 6 (2.52) | 105 (44.12) | 127 (53.36) |
|  | **T2_FA** | 0 (0.00) | 0 (0.00) | 12 (5.04) | 92 (38.66) | 134 (56.30) |
| **Time Spent with the Doctor (TSWD)** | **T1_TSWD** | 0 (0.00) | 0 (0.00) | 6 (2.52) | 105 (44.12) | 127 (53.36) |
|  | **T2_TSWD** | 0 (0.00) | 2 (0.84) | 6 (2.52) | 112 (47.06) | 118 (49.58) |
| **Accessibility and Convenience (AC)** | **T1_AC** | 0 (0.00) | 0 (0.00) | 11 (4.62) | 165 (69.33) | 62 (26.05) |
|  | **T2_AC** | 0 (0.00) | 0 (0.00) | 11 (4.62) | 132 (55.46) | 95 (39.92) |

Abbreviations: T1, first time point; T2, second time point; GS, General Satisfaction; TQ, Technical Quality; IM, Interpersonal Manner; Com, Communication; FA, Financial Aspects; TSWD, Time Spent with Doctor; AC, Accessibility and Convenience.

The above table depicts the frequency and the corresponding percentages of the different scores assigned by participants overall and for each PSQ subscale per time point. No trends are observed between the two time points regarding scores 1-3. However, a shift occurs from score 4 to 5, when comparing time point 2 with time point 1 (i.e., frequency of participants indicating 5 increases at time point 2 as compared to the frequency who indicated 4).

^a^The mean scores were calculated as the average score of the corresponding questions of the modified Patient Satisfaction Questionnaire (PSQ-18). The overall score is comprised of 18 responses while each subscale contains variable numbers of responses (see eTable 1). The final mean score was rounded to the nearest integer (if the decimal part is less than 5, then the score is rounded down; otherwise, it is rounded up).

^b^The percentages were calculated over the 344 participants.

^c^The percentages were calculated over the 238 telemedicine participants.

**Supplementary Table S3:** Model Covariates

| **Modeling code** | **Definition** | **Categories** |
| --- | --- | --- |
| A | Study arm | Referral (reference level) |
|  |  | Telemedicine |
| T | Time point | Initial provider encounter (reference level) |
|  |  | Sustained virological response encounter |
| G | Gender | Male (reference level) |
|  |  | Female |
| R | Race | White (reference level) |
|  |  | Black/African-American |
|  |  | Other race |
| Ag | Age | Continuous variable – no categories |
| E | Ethnicity | Non-Hispanic (reference level) |
|  |  | Hispanic |
| CC | Comorbid condition | No comorbid condition or unsure (reference level) |
|  |  | Anxiety and/or depression |
|  |  | Other co-morbid conditions |
| CM | Combined monthly income | Middle income ($834-$1249, reference level) |
|  |  | Higher income ($1250 or more) |
|  |  | Low income ($0-$833) |
| RT | Residence type | Private residence (reference level) |
|  |  | Other |
| H | Highest education level | Less than or equal to 11th grade (reference level) |
|  |  | 12th grade / completed high school or received GED / Post high school training |
|  |  | Some college/College graduate or Postgraduate |

Abbreviations: GED, general educational development.

**Figure legends**

**Supplementary Figure S1:** Interpretation of transcripts of individuals experiencing facilitated telemedicine for hepatitis C virus treatment.

**Supplementary Figure S2:** These graphs illustrate the barplots of the differences in the mean scores between the two time points (T2-T1) for all (#344) and telemedicine (#238) participants, overall (1^st^ bar) and per PSQ subscale (remaining bars). Abbreviations: T1, first time point; T2, second time point; GS, General Satisfaction; TQ, Technical Quality; IM, Interpersonal Manner; Com, Communication; FA, Financial Aspects; TSWD, Time Spent with Doctor; AC, Accessibility and Convenience.

**Supplementary Figure S3:** These graphs illustrate the boxplots of the scores in the 7 PSQ subscales per arm and per time point for all (#344) participants. The vertical lines issuing from the boxes extend from the minimum to maximum values, the length of the boxes represents the interquartile range, the symbols (circles) in the boxes represent the means, whereas the horizontal lines in the boxes represent the medians. Abbreviations: T1, first time point; T2, second time point; GS, General Satisfaction; TQ, Technical Quality; IM, Interpersonal Manner; Com, Communication; FA, Financial Aspects; TSWD, Time Spent with Doctor; AC, Accessibility and Convenience.

**Supplementary Figure S1:** Interpretation of transcripts of individuals experiencing facilitated telemedicine for hepatitis C virus treatment

Interviews conducted, recorded, transcribed, verified for accuracy and de-identified for analysis.

Hermeneutic interpretation


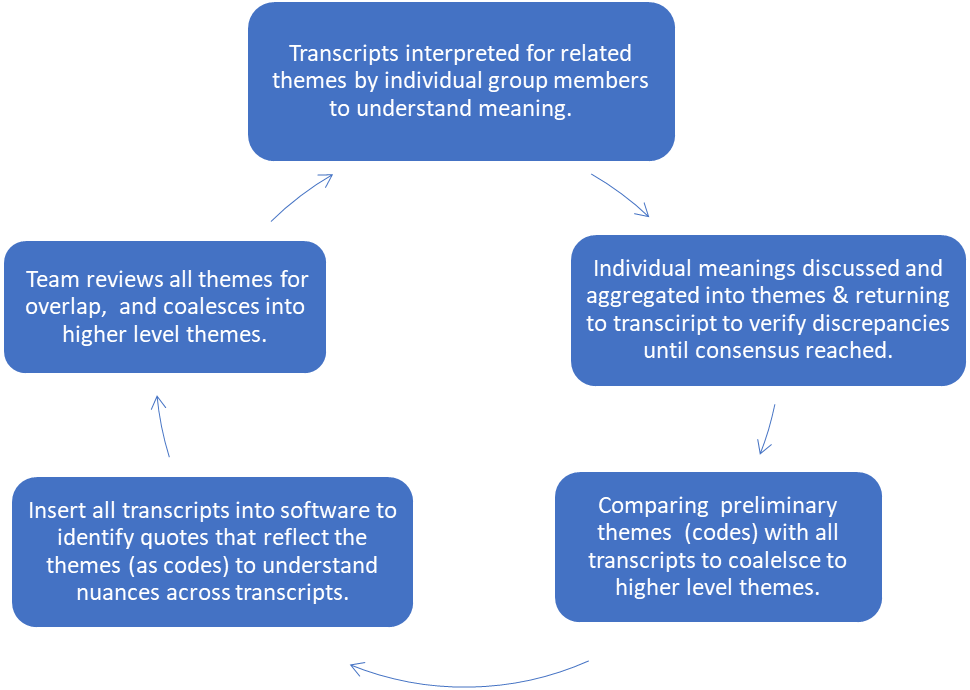


The figure above illustrates the hermeneutic phenomenological approach followed to understand and code the meanings of hepatitis C virus treatment integrated into an opioid treatment program.

**Supplementary Figure S2:** Barplots of the Difference (T2-T1) in the Mean Scores for all (#344) and telemedicine (#238) participants

| **** |
| --- |
| **** |

**Supplementary Figure S3:** Boxplots of the Scores in the 7 subscales per arm and time point for all (#344) participants

| 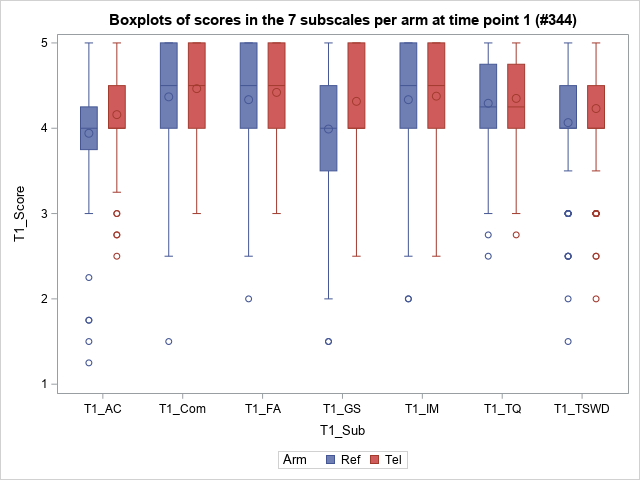 |
| --- |
| 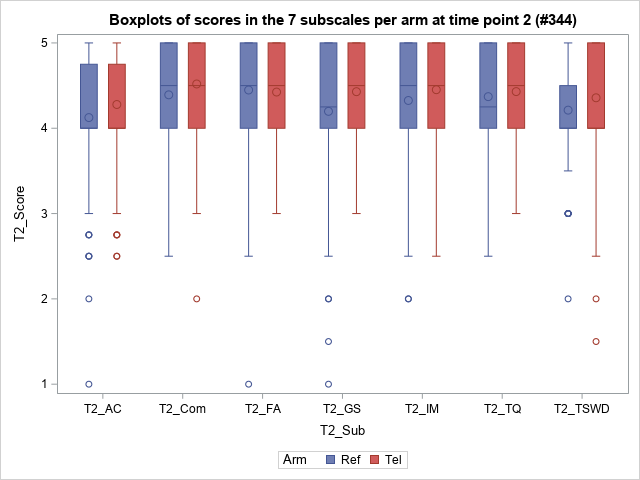 |

**References**

1. Marshall GN, Hays RD, The patient satisfaction questionnaire short-form (PSQ-18). Santa Monica, CA: RAND, 1994.

2. Kiernan, K., Insights into Using the GLIMMIX Procedure to Model Categorical Outcomes with Random Effects Paper SAS2179-2018 2018: 1–23.

3. Kiernan K, Tao J, Gibbs P. Tips and Strategies for Mixed Modeling with SAS/STAT Procedures. Paper SAS6403-2016 2016: 1-21.

4. Peterson B, Harrell FE. Partial proportional odds models for ordinal response variables. J R Stat Soc Ser C Appl Stat 1990; 39: 205-217.

5. SAS Institute, SAS/STAT 14.1 User's Guide The NLMIXED Procedure.Cary, NC: SAS Institute Inc., 2015.

6. Agresti A. Categorical Data Analysis 2nd ed. Hoboken, NJ: Wiley and Sons, 2002.

7. Talal AH, McLeod A, Andrews P, et al. Patient reaction to telemedicine for clinical management of hepatitis C virus integrated into an opioid treatment program. Telemed E-Health 2019; 25:791-801.

8. Social Security Disability Administration. The Faces and Facts of Disability 2018 Accessed: Febuary 25, 2022; Available from: <https://www.ssa.gov/disabilityfacts/facts.html>.

9. Dibley L, Dickerson S, Duffy M, et al., Doing hermeneutic phenomenological research: A practical guide. Thousand Oaks, CA: SAGE, 2020.

10. Heidegger M. Being and time. New York, NY: Harper and Row, 1962.

11. Gadamer H.-G. Truth and method (Revised 2nd ed.). New York, NY: Continuum, 1998.

12. Smythe EA, Ironside PM, Sims SL, et al. Doing Heideggerian hermeneutic research: A discussion paper. Int J Nurs Stud 2008; 45: 1389-1397.

13. Ironside PM, Hermeneutics. In: Fitzpatrick JJ, Kazer MW, eds. Encyclopedia of nursing research. New York, NY: Springer Publishing Company, 2017.

14. Gibbs GR, Analyzing qualitative data.Thousand Oaks, CA: Sage Publications, Ltd., 2007.

15. De Witt L, Ploeg J. Critical appraisal of rigour in interpretive phenomenological nursing research. J Adv Nurs 2006;55: 215-229.

16. Sparck-Jones KA. Statistical interpretation of term specificity and its application in retrieval. J Doc 2004; 60: 493-502.
